# Methods for Addressing Missing Patient-Reported Outcome Data in Longitudinal Clinical Trials: A Secondary Analysis of the Frequent Hemodialysis Network Trials

**DOI:** 10.1177/20543581261491677

**Published:** 2026-09-28

**Authors:** Victoria Zdunek, Guangyong Zou, Amit X. Garg, Pavel S. Roshanov

**Affiliations:** 1 Department of Epidemiology and Biostatistics, Schulich School of Medicine and Dentistry, 70384 Western University, London, ON, Canada; 2 Robarts Research Institute, 70384 Western University, London, ON, Canada; 3 Division of Nephrology, Department of Medicine, Schulich School of Medicine and Dentistry, 70384 Western University, London, ON, Canada; 4 London Health Sciences Research Institute, London, ON, Canada; 5 10033 London Health Sciences Centre, London, ON, Canada; 6 33493 Population Health Research Institute, Hamilton, ON, Canada

**Keywords:** Patient-reported outcome measures, hemodialysis, longitudinal trials, missing data, win probability

## Abstract

**Background:**

Patient-reported outcomes from longitudinal clinical trials on hemodialysis typically have missing data, yet there is a lack of empirical evidence to guide appropriate analysis and interpretation.

**Objective:**

To compare approaches for analysis and interpretation of patient-reported outcomes in the Frequent Hemodialysis Network (FHN) Trials.

**Design:**

Secondary analysis of two multicentre randomized controlled trials.

**Setting:**

Hemodialysis centres and home hemodialysis programs in North America.

**Patients:**

Participants in the FHN Daily (n = 245) and Nocturnal (n = 87) Trials.

**Measurements:**

RAND-36 physical health composite scores at baseline, months 4 and 12.

**Methods:**

Complete case analysis (CCA), mixed model for repeated measures (MMRM), and multiple imputation (MI) were used to estimate mean differences in scores at Month 12. To relax the normality assumption and enhance interpretation of mean differences, rank-based analyses were performed to estimate the win probability (WinP) that a treated participant has a better score than (or wins over) a control participant at Month 12.

**Results:**

Data complete at both post-randomization timepoints were available for 74% to 75% of participants. In the Daily Trial, estimates of Month 12 mean difference (95% CI) were 3.04 (0.66 to 5.42, p = 0.012) by MMRM, 2.97 (0.42 to 5.52, p = 0.023) by MI, and 2.76 (0.31 to 5.21, p = 0.027) by CCA. Estimates of WinP were 0.59 (0.52 to 0.65, p = 0.007) by MMRM and 0.59 (0.52 to 0.65, p = 0.011) by CCA. In the Nocturnal Trial, treatment effects were small, imprecise, and not statistically significant across all methods.

**Limitations:**

This was a secondary analysis and rank-based analyses were exploratory.

**Conclusions:**

Approaches that retained participants with partially observed data can be more efficient than complete-case analyses. Win probability analyses provided an alternative to mean differences in patient-reported outcome measure scores.

## 1. Introduction

More than 50,000 Canadians receive maintenance dialysis, many of whom rely on thrice-weekly in-centre hemodialysis for survival.^1^ While life-sustaining, patients experience substantial burdens, including fatigue, dietary and fluid restrictions, limited mobility, and psychological distress.^1-5^ Kidney failure and its underlying comorbidities also contribute to these burdens, and their effects cannot be fully separated from those of dialysis outside clinical trials.^1,4^ As a result, health-related quality of life remains persistently low in this population, often lower than that observed in other chronic conditions.^4,5^ Persistent impairments in physical, mental, and social functioning have prompted international initiatives to establish health-related quality of life as a core outcome in kidney medicine trials, clinical decision-making, and guidelines.^6,7^

Randomized controlled trials commonly assess health-related quality of life with validated patient-reported outcome measures (PROMs); the RAND 36-Item Health Survey (RAND-36) is among the most frequently used, yielding two composite scores: Physical Health Composite (PHC) and Mental Health Composite (MHC).^8^ In hemodialysis populations, PHC scores predict hospitalization and mortality,^9-11^ while MHC scores are associated with depression and mental health-related hospitalizations.^9-11^ Despite their importance, patient-reported outcomes in longitudinal dialysis trials are often incomplete due to hospitalization, comorbidities, discontinuation of dialysis treatment, withdrawal from study follow-up, or death.^12^ Such events are influenced by patient health during follow-up and may occur independently of intervention effects, and they are typically related to patient characteristics (e.g., baseline health status or treatment assignment), making the assumption of missing completely at random implausible.^13,14^ When missingness depends on observed characteristics such as previous measurements, complete-case analysis (CCA) can yield biased estimates and reduced statistical efficiency.^13,15,16^

Mean differences in change scores between treatment groups at follow-up times remain the predominant treatment-effect metric in dialysis trials when baseline and follow-up measurements are available. However, patient-reported outcome distributions are often skewed, bounded, affected by floor or ceiling effects, and contain outliers.^17-19^ These features can challenge the parametric assumptions and complicate clinical interpretation, especially when a minimally clinically important difference is unavailable.^17-19^ As an alternative, we also considered a rank-based approach to estimate win probability (WinP), the probability that a randomly selected patient in the treatment has a better score than a patient in the control group.^20-22^

The Frequent Hemodialysis Network (FHN) Daily and Nocturnal Trials provide an opportunity to evaluate these issues. Both trials compare more frequent hemodialysis schedules with conventional thrice-weekly hemodialysis and collected clinical and patient-reported outcomes, including RAND-36 health-related quality of life scores.^23,24^ Participant-reported surveys were collected by a central survey centre whose staff were blinded to treatment assignment. While the Daily Trial reported significant mean improvements in PHC, the Nocturnal Trial did not after covariate adjustment.^23-25^ Both trials were subject to considerable missing data by 12 months, arising from death, treatment discontinuation, and loss to follow-up. Death represents missing data that could not be collected; loss to follow-up and survey noncompletion represent potentially avoidable missing data. Together, these patterns raise uncertainty about the robustness of the original findings.

Our primary objective was to compare how treatment-effect interpretation changes across these methods when PROM data are incomplete. Specifically, we used data from the FHN Daily and Nocturnal Trials to estimate mean differences in PHC scores at 4- and 12-months using three approaches for handling missing data: CCA, mixed models for repeated measures (MMRM), and multiple imputation (MI). Without relying on normality assumptions, we also estimated the WinP as a complement to mean differences.

## 2. Methods

### 2.1. FHN Dataset Characteristics

We analyzed individual participant data from the FHN Daily and Nocturnal Trials, two parallel, multicentre randomized controlled trials conducted across North America. The Daily Trial randomized patients treated with in-centre hemodialysis to conventional (3 times/week) or frequent (6 times/week) schedules.^23^ The Nocturnal Trial enrolled patients receiving home hemodialysis and compared conventional dialysis with frequent nocturnal dialysis (6 times/week, with longer sessions).^24,25^ Both trials collected PROMs at baseline, 4 months, and 12 months, including the RAND-36 PHC score.^23-25^ Analyses followed original randomization, with no modification of treatment arms or protocols. Full trial protocols and primary results have been described elsewhere.^23-25^ The datasets analyzed during the current study are available in the NIDDK Central Repository, (Daily: https://repository.niddk.nih.gov/study/44, Nocturnal: https://repository.niddk.nih.gov/study/168).

The trials were analyzed separately to preserve differences in treatment modality and setting. A consistent modeling framework and covariate structure was applied where applicable. Analyses were restricted to participants with non-missing baseline PHC scores. Models adjusted for baseline PHC and dialysis centre, consistent with original trial analyses.^23,24^

### 2.2. Missing Data

#### 2.2.1. Missing Data Types

Missing data are common in longitudinal PROM-based randomized controlled trials, where attrition may reflect illness severity, respondent fatigue, or disengagement due to declining health.^13,15^ We characterized missing PHC scores by both structure and temporal pattern.

Two structural types of missingness were considered. Item-level missingness refers to the absence of responses within a multi-item scale; this can compromise score derivation, reduce internal consistency, and weaken the psychometric properties of the instrument.^14,26,27^ Wave-level missingness refers to the absence of an entire measurement occasion (e.g., a missing PHC score at the 4-month visit).^14,26,27^ While the RAND-36 permits item-level scoring approaches, the FHN datasets available for download included only subscale and pre-aggregated composite scores, not individual item responses. As such, this analysis focused exclusively on wave-level missingness.

We also classified temporal patterns. A monotone pattern occurs when a missing value at one timepoint is followed by missing values at all subsequent timepoints (e.g., observed at baseline and 4 months but missing at 12 months)^14,27^; in practical terms, once a participant stops providing PHC data, they do not return at later assessments. A non-monotone pattern involves intermittent missingness (e.g., missing the 4 month PHC score but observed again at 12 months).^14,27^ These distinctions have analytic implications. Some methods (e.g., standard implementation of MI) assume monotonicity or behave differently depending on whether missingness reflects sustained dropout or intermittent nonresponse.^14,27^ However, imputation approaches based on fully conditional specification (chained equations) do not require monotone missingness and can accommodate intermittent nonresponse directly; we retain the distinction here because it informs our characterization of dropout patterns and their implications for the plausibility of missing at random (MAR) versus missing completely at random assumptions. Recognizing these structures informs both diagnostics and interpretation of robustness across methods.

#### 2.2.2. Missing Data Mechanisms

Rubin’s framework classifies missingness into three categories: missing completely at random, missing at random, and missing not at random, each with distinct implications for bias, efficiency, and validity.^14^

Missing completely at random occurs when the probability of missingness is unrelated to observed and unobserved data.^14^ This assumption is rarely plausible in dialysis populations, because PROMs are often missing due to hospitalization, poor health status, treatment burden, or clinical events such as kidney transplantation that lead to discontinuation of dialysis and difficulty obtaining follow-up responses.^13^ Thus, MAR assumption is more plausible if the variables related to data missing are accounted for in data analysis.

Missing not at random arises when missingness depends on the unobserved outcome even after conditioning on observed covariates and outcomes.^14^ For instance, if participants with the lower PHC score at Month 4 were systematically more likely to drop out and the data at Month 4 were not accounted for in the analysis, then the underlying assumption for estimating treatment effect at Month 12 is missing not at random. Analyses under missing not at random generally require making assumptions based on external information, e.g., assuming dropout participants in the treatment group behaved more like those in the control group. Such analyses are usually performed as sensitivity analyses.^26^

Given the clinical context of the FHN trials, the missing completely at random assumption was considered implausible, as attrition was likely related to patients’ health status and treatment experience. We therefore investigated whether MAR could serve as a reasonable working assumption. To do so, we examined dropout patterns and correlations across observed PHC scores over time. These diagnostics can indicate whether observed PHC values contain information about later PHC values, which is useful for longitudinal modeling, but cannot determine whether the missingness mechanism is MAR.^13,15,16^ We also note that auxiliary variables (i.e., predictors of PHC and/or of missingness, such as recent hospitalization, comorbidity burden, lab indices, adherence markers) were not incorporated into our primary models; this constraint may reduce the validity of analysis based on the assumption of MAR.

#### 2.2.3. Missing Data Methods

We applied three analytic strategies to address missing PHC scores: complete-case analysis, mixed model for repeated measures, and multiple imputation with a focus on estimating treatment effects at months 4 and 12. These approaches are summarized in Supplementary Table S1. Complete-case linear regression models were fitted separately at each follow-up timepoint and adjusted for treatment group, baseline PHC score, baseline PHC-by-treatment interaction, and dialysis centre. MMRM modeled observed follow-up PHC scores at months 4 and 12 as repeated outcomes and included fixed effects for timepoint, treatment group, their interaction, baseline PHC score and its interaction with treatment group, and dialysis centre. MI imputed missing follow-up PHC scores before estimating the same timepoint-specific treatment effects. Analyses were conducted using Stata version 19.5 and R version 4.4.3.

Complete-case analysis included only participants with baseline PHC and observed PHC scores at the timepoint analyzed. Separate linear regression models were fitted at months 4 and 12, adjusting for treatment group, baseline PHC score and its interaction with treatment group, and dialysis centre. This approach does not require participants to have observed PHC scores at both follow-up timepoints; however, participants missing the target follow-up outcome were excluded from that timepoint-specific analysis, and observed outcomes at other follow-up timepoints were not used to inform the estimate. This analysis is simple to implement, but it relies on the strong assumption that data are missing completely at random. This assumption is unlikely in the FHN trials, where attrition was related to health status. When violated, CCA may introduce bias and reduce power by excluding participants with partial data.^15^ For example, if participants with lower baseline PHC were disproportionately likely to miss the 12-month assessment, CCA could overestimate physical health outcomes.^13,14^

The mixed model for repeated measures is a likelihood-based method that incorporates all available outcome data and retains participants with partial follow-up, thereby increasing statistical efficiency.^26,28^ Models included fixed effects for timepoint, treatment group, and their interaction; baseline PHC (with interaction by treatment group); and dialysis centre. Participants were specified as the repeated-measures units, and an unstructured residual covariance matrix was used to account for correlations among PHC scores within a participant. Estimation was performed using restricted maximum likelihood with Kenward-Roger adjustments to degrees of freedom. Under MAR, likelihood-based repeated-measures models yield unbiased estimates and valid standard errors when the model is correctly specified.^26,28^

Multiple imputation addresses missing data by generating multiple plausible values for missing observations and combining results across completed datasets.^14,15,26^ We used multiple imputation by chained equations for PHC scores with predictive mean matching to impute PHC scores at months 4 and 12. Missing follow-up PHC values were imputed jointly from baseline PHC, treatment group, and dialysis centre, with observed PHC at one follow-up informing imputation of the other. For each missing value, five donor cases with similar predicted means were identified, and one observed value was randomly drawn to replace it. After a 20-iteration burn-in to ensure convergence, 100 imputed datasets were created. Each completed dataset was analyzed using a linear regression model including timepoint, treatment group, their interaction, baseline PHC score, baseline PHC-by-treatment interaction, and dialysis centre; estimates were combined using Rubin’s rules. MI is particularly advantageous when MAR is plausible and relevant predictors of missingness are available.^14,16^

### 2.3. Secondary Analysis: WinP

The mean-based approaches rely on parametric modeling assumptions, including approximate normality, and treat PHC scores as continuous outcomes with meaningful units. As a complementary analysis, we adopted a distribution-free method to estimating the probability that a randomly selected participant from the treatment group reports a better outcome than a randomly selected participant from the control group.^20,21^ This probability has been termed win probability (WinP).^20,21,29^ As a rank-based measure, it is applicable to binary, ordinal, and continuous data. A WinP of 0.50 indicates no difference between groups, while a WinP of 0.65 implies a 65% probability that a patient randomized to more frequent dialysis would report better physical health-related quality of life than one receiving conventional dialysis. For normally distributed data, WinP is a one-to-one function of the standardized mean difference, also known as Cohen’s effect size. Standardized mean differences of 0.2, 0.5, and 0.8 correspond to WinP values of 0.56, 0.64, and 0.71, respectively.^29^

As detailed elsewhere,^22^ estimation of WinP in longitudinal RCTs begins with using ranks to convert each observation at a timepoint into a win fraction, reflecting the proportion of times that the observation is better than every observation in the comparison group at the given timepoint. Then, we replaced the PHC scores in linear regression models by the win fractions as the outcomes to obtain the point and variance estimates of WinP. In our case, we first obtained win fractions at baseline and Months 4 and 12, followed by applying the MMRM model that included fixed effects for timepoint, treatment group, and their interaction, as well as baseline win fraction and baseline PHC score (each interacted with treatment group), and dialysis centre. An unstructured covariance matrix was specified separately for each treatment arm to account for within-person correlation. Estimation was performed using restricted maximum likelihood with Kenward-Roger adjustments to degrees of freedom.

We did not apply MI to WinP analyses. Simulation studies have shown that imputing raw PROM scores prior to rank transformation can distort the WinP estimand, impose unnecessary model dependence, and yield overly conservative inference (e.g., empirical rejection rates near 2.5% at a nominal 5%), leading to reduced statistical power. For these reasons, MMRM applied directly to win fractions was preferred, as it preserves the nonparametric nature of the WinP and makes use of all observed data under the MAR assumption without imputation. This approach is consistent with recent methodological work in which a multivariate mixed-model framework was used to estimate endpoint-specific win probabilities under missing data.^30^

#### 2.3.1. Sensitivity Analysis

For sensitivity analyses, we used a hierarchical composite win fraction outcome that combined death and PHC change, consistent with the original FHN coprimary rank-based approach. Participants who died before follow-up were ranked by survival time, with shorter survival indicating worse outcome. Survivors were ranked by change in PHC from baseline, and all deaths were ranked below all survivors. As PHC missingness due to death is unlikely to satisfy standard MAR assumptions, this composite directly incorporates death as the worst outcome while retaining PHC change among survivors. We then analyzed these participant-level summaries using the same CCA and MMRM approaches described above.

## 3. Results

### 3.1. Analytic Samples and Baseline Characteristics

The analytic sample included 245 participants in the Daily Trial (125 randomized to frequent dialysis and 120 to conventional dialysis across 11 centres) and 87 participants in the Nocturnal Trial (45 randomized to frequent nocturnal dialysis and 42 to conventional dialysis across 8 centres).

Baseline PHC scores were balanced across arms (Daily: 38.1 vs. 37.8; Nocturnal: 37.3 vs. 37.5). Baseline variability was somewhat greater in the frequent dialysis arms (Daily SD = 11.7 vs Conventional SD = 9.7; Nocturnal SD = 9.6 vs Conventional SD = 10.1), consistent with expectations in smaller samples.

Score distributions differed between trials (Figures 1 and 2). Distributions were approximately symmetric in the Daily Trial, but more clearly non-normal in the Nocturnal Trial, with skewness, heavier tails, and outliers. Such distributional features may challenge parametric mean-based analyses and motivate complementary rank-based analyses.

**Figure 1.**
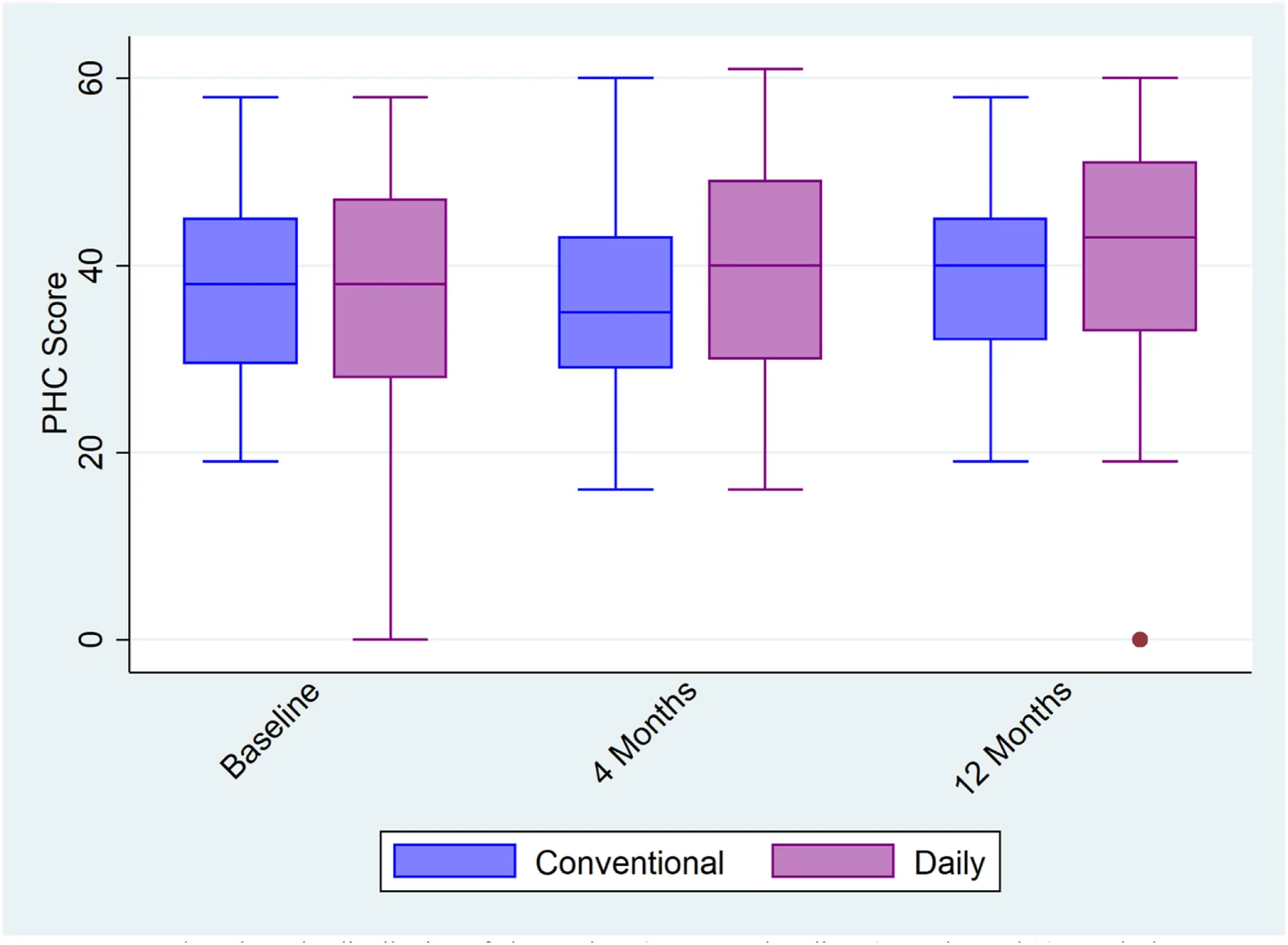
PHC Scores at Each Timepoint by Treatment Group in the FHN Daily Trial. **Footnote:** Boxplots show the distribution of observed PHC scores at baseline, 4 months, and 12 months by randomized treatment group. The horizontal line within each box represents the median, boxes represent the interquartile range, whiskers represent the non-outlying range, and points represent outliers. Higher PHC scores indicate better physical health-related quality of life. Abbreviations: FHN, Frequent Hemodialysis Network; PHC, Physical Health Composite

**Figure 2.**
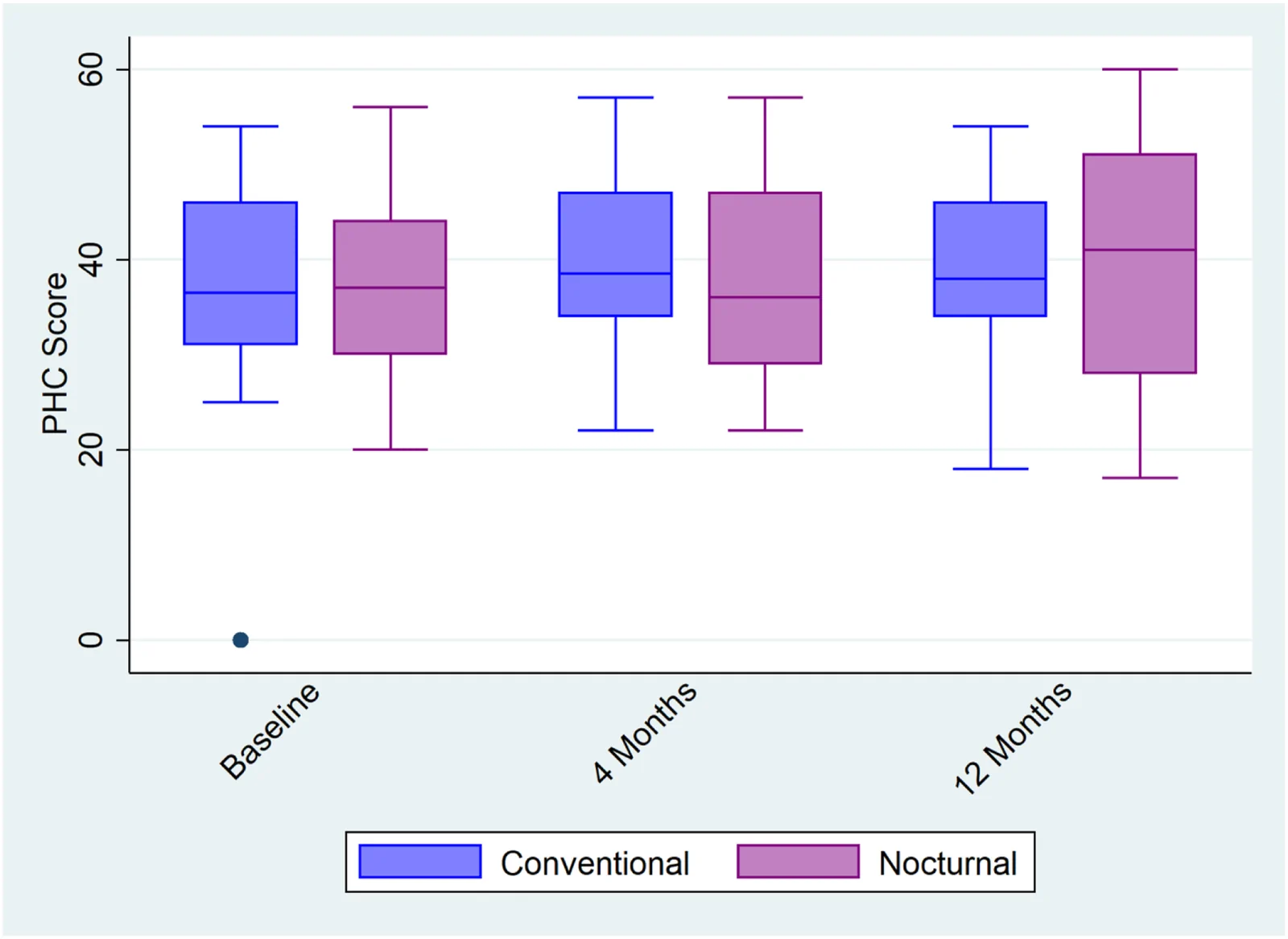
PHC Scores at Each Timepoint by Treatment Group in the FHN Nocturnal Trial. **Footnote:** Boxplots show the distribution of observed PHC scores at baseline, 4 months, and 12 months by randomized treatment group. The horizontal line within each box represents the median, boxes represent the interquartile range, whiskers represent the non-outlying range, and points represent outliers. Higher PHC scores indicate better physical health-related quality of life. Abbreviations: FHN, Frequent Hemodialysis Network; PHC, Physical Health Composite

### 3.2. Missing Data Patterns

Attrition increased over time in both trials (Table 1). In the Daily Trial, 13.5% of overall PHC scores were missing at 4 months and 22.0% at 12 months. The Nocturnal Trial showed similar dropout, with 6.9% missing at 4 months and 21.8% at 12 months.

**Table 1.** Summary of PHC Scores by Timepoint

Timepoint	Treatment Arm	Mean PHC	SD	Number of Observed PHC Scores (n)	Missing PHC Scores (%)
**FHN Daily**					
Baseline	Conventional	38.05	9.73	120	0.0
​	Daily	37.81	11.67	125	0.0
​	Overall	37.93	10.74	245	0.0
4 Months	Conventional	36.00	9.68	98	18.3
​	Daily	39.84	11.31	114	8.8
​	Overall	38.07	10.74	212	13.5
12 Months	Conventional	38.67	9.46	86	28.3
​	Daily	41.28	11.46	105	16.0
​	Overall	40.10	10.66	191	22.0
**FHN Nocturnal**					
Baseline	Conventional	37.31	10.12	42	0.0
​	Nocturnal	37.49	9.60	45	0.0
​	Overall	37.40	9.80	87	0.0
4 Months	Conventional	39.93	9.17	40	4.8
​	Nocturnal	38.61	10.53	41	8.9
​	Overall	39.26	9.84	81	6.9
12 Months	Conventional	39.36	9.18	33	21.4
​	Nocturnal	40.46	12.54	35	22.2
​	Overall	39.93	10.97	68	21.8

Abbreviations: PHC, Physical Health Composite; SD, standard deviation.

Most missingness followed a monotone pattern, with dropout dominating in both trials (Tables 2 and 3). In the Daily Trial, 13% of participants dropped out after 4 months and 9% after baseline, with only 4% showing intermittent missingness. The Nocturnal Trial exhibited similar trends, with 18% dropout after 4 months, 3% after baseline, and 3% intermittent patterns. Complete data across all three timepoints were available for 74 to 75% of participants in both trials.

**Table 2.** Wave-Level Missing Data Patterns for PHC Scores by Treatment Group for the FHN Daily Trial

Missingness Pattern	Baseline	4 Months	12 Months	Overall (%)	Conventional (%)	Daily (%)
Complete	✓	✓	✓	74	67	81
Dropout after 4 months	✓	✓	✗	13	15	10
Dropout after baseline	✓	✗	✗	9	13	6
Intermittent	✓	✗	✓	4	5	3

✓ = observed (non-missing); ✗ = missing. Percentages are rounded and may not sum to 100%. All participants had complete baseline PHC scores by design.

**Table 3.** Wave-Level Missing Data Patterns for PHC Scores by Treatment Group for the FHN Nocturnal Trial

Missingness Pattern	Baseline	4 Months	12 Months	Overall (%)	Conventional (%)	Nocturnal (%)
Complete	✓	✓	✓	75	76	73
Dropout after 4 months	✓	✓	✗	18	19	18
Dropout after baseline	✓	✗	✗	3	2	4
Intermittent	✓	✗	✓	3	2	4

✓ = observed (non-missing); ✗ = missing. Percentages are rounded and may not sum to 100%. All participants had complete baseline PHC scores by design.

Within-person PHC scores were moderately to strongly correlated across timepoints, as shown in Figures 3 and 4 (Daily Trial: r = 0.59 to 0.74; Nocturnal Trial: r = 0.60 to 0.72). These correlations indicate that observed PHC scores contained information about later PHC values, supporting longitudinal models that incorporate repeated observed outcomes. However, they should not be interpreted as proof that the missingness mechanism was MAR. The combination of monotone dropout and selective attrition further highlights the vulnerability of CCA to biased estimation, particularly when participants with poorer baseline health are disproportionately excluded.

**Figure 3.**
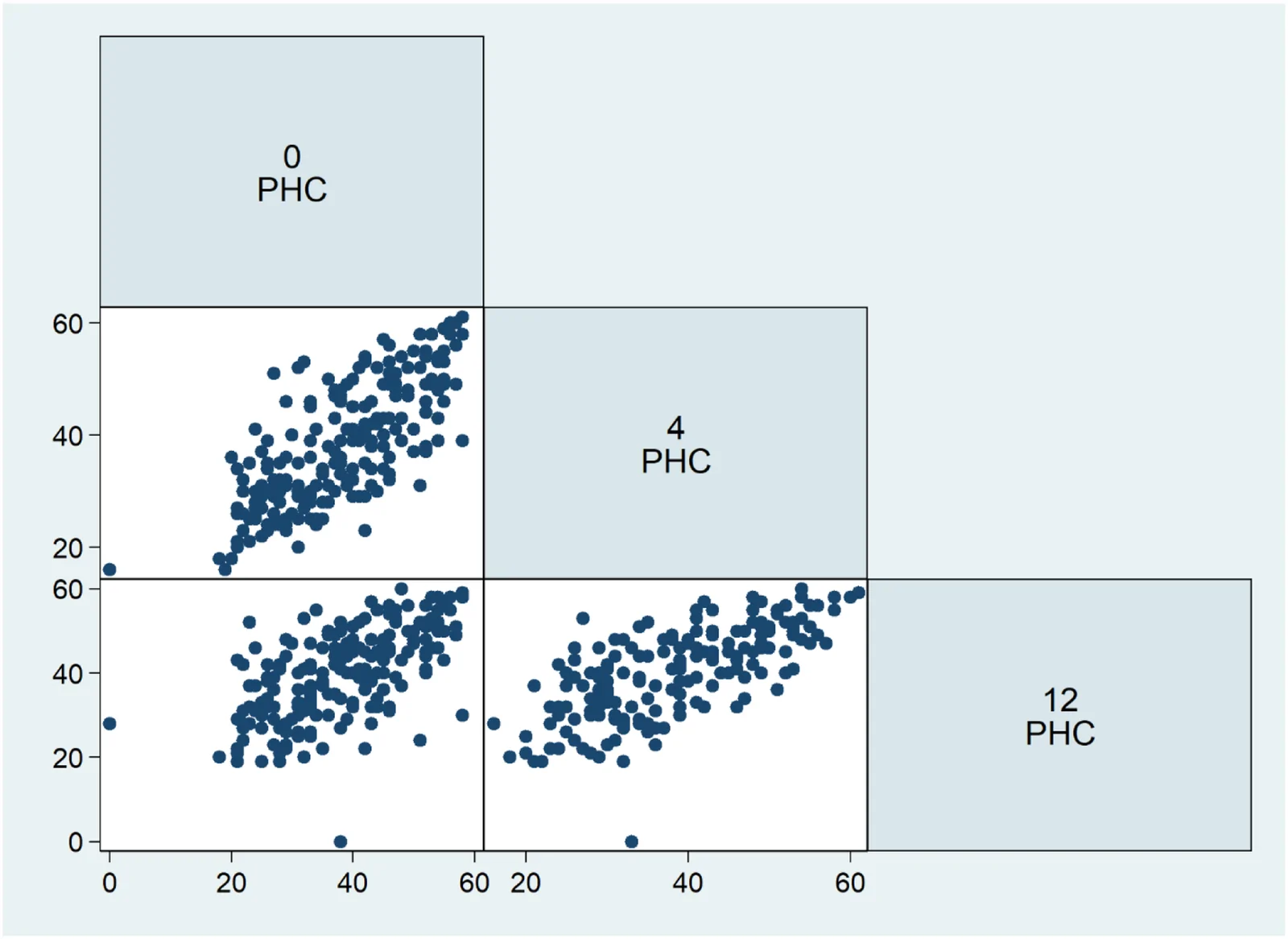
Pairwise Correlations of Observed PHC Scores Over Time in the FHN Daily Trial. **Footnote:** Each panel compares observed PHC scores for the same participants at two timepoints. Both axes use the PHC score scale, with higher values indicating better physical health-related quality of life; repeated 0-60 scales reflect the different timepoint pairs being compared. Points represent participants with PHC observed at both timepoints in the pair, and correlations summarize within-person consistency over time. Abbreviations: FHN, Frequent Hemodialysis Network; PHC, Physical Health Composite

**Figure 4.**
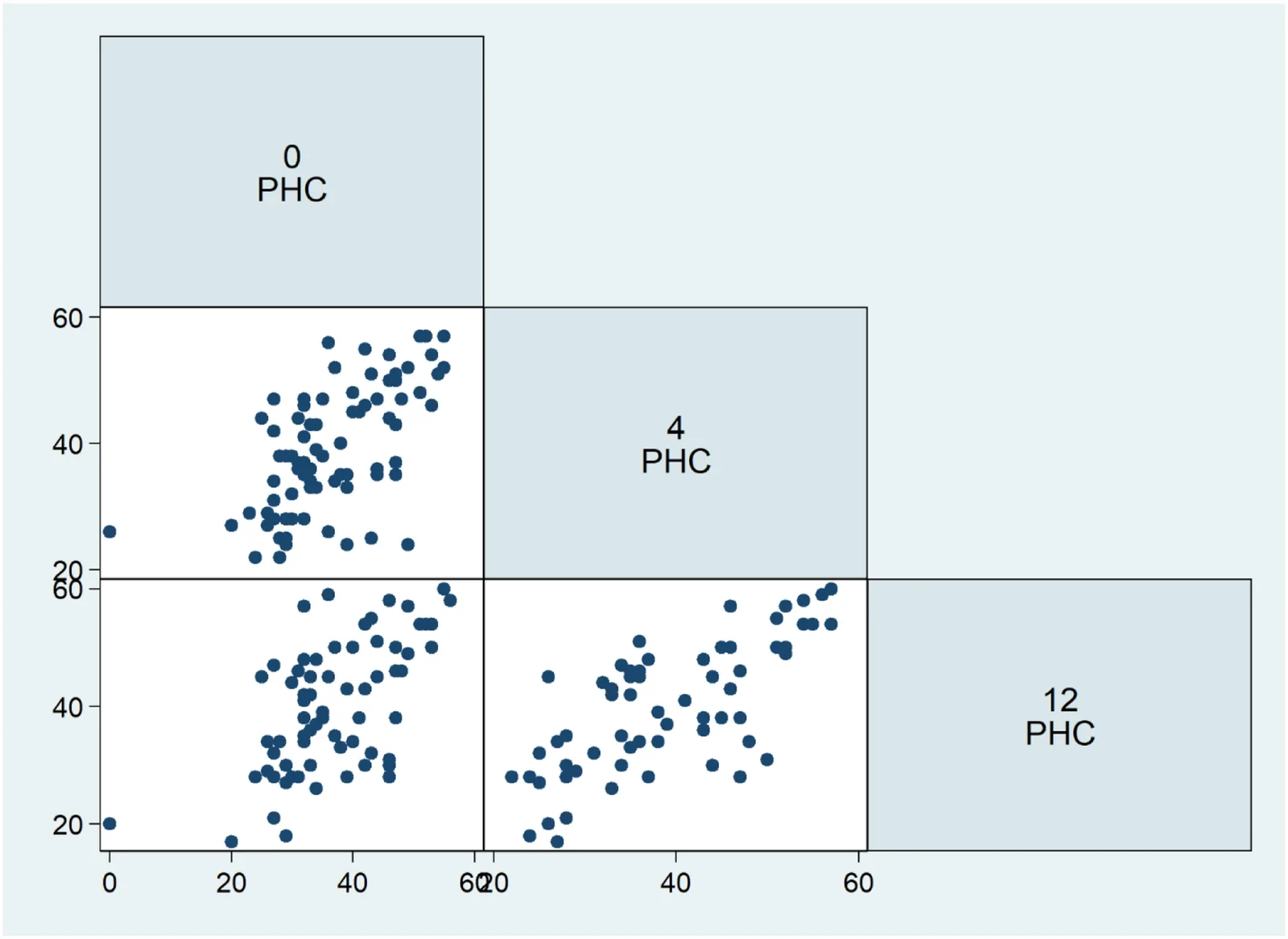
Pairwise Correlations of Observed PHC Scores Over Time in the FHN Nocturnal Trial. **Footnote:** Each panel compares observed PHC scores for the same participants at two timepoints. Both axes use the PHC score scale, with higher values indicating better physical health-related quality of life; repeated 0-60 scales reflect the different timepoint pairs being compared. Points represent participants with PHC observed at both timepoints in the pair, and correlations summarize within-person consistency over time. Abbreviations: FHN, Frequent Hemodialysis Network; PHC, Physical Health Composite

### 3.3. Treatment Effect Estimates

#### 3.3.1. Mean Differences in Scores

In the Daily Trial, treatment effect estimates were similar across analytic approaches (Table 4). At 4 months, all three approaches estimated higher PHC scores with frequent dialysis: 3.92 points with CCA (95% CI: 2.00 to 5.85; p < 0.001), 3.95 points with MMRM (95% CI: 2.05 to 5.85; p < 0.001), and 3.92 points with MI (95% CI: 1.50 to 6.34; p = 0.002). At 12 months, estimated benefits were smaller but remained statistically significant: 2.76 points with CCA (95% CI: 0.31 to 5.21; p = 0.027), 3.04 points with MMRM (95% CI: 0.66 to 5.42; p = 0.012) and 2.97 points with MI (95% CI: 0.42 to 5.52; p = 0.023).

**Table 4.** Mean Differences in PHC Scores Over Time in the FHN Trials

Timepoint	Method (n)	Effect Estimate (95% CI)	95% CI Width	p-value
**Daily Trial**				
4 months	CCA (212)	3.923 (1.995 to 5.850)	3.855	<0.001
​	MMRM (245)	3.949 (2.049 to 5.848)	3.799	<0.001
​	MI (245)	3.920 (1.497 to 6.343)	4.846	0.002
12 months	CCA (191)	2.760 (0.312 to 5.208)	4.896	0.027
​	MMRM (245)	3.040 (0.657 to 5.424)	4.767	0.012
​	MI (245)	2.968 (0.418 to 5.518)	5.100	0.023
**Nocturnal Trial**				
4 months	CCA (81)	-0.644 (-4.134 to 2.845)	6.979	0.714
​	MMRM (87)	-0.695 (-4.113 to 2.723)	6.836	0.690
​	MI (87)	-1.063 (-5.284 to 3.158)	8.442	0.620
12 months	CCA (68)	1.257 (-2.970 to 5.484)	8.454	0.554
​	MMRM (87)	1.263 (-2.731 to 5.257)	7.988	0.536
​	MI (87)	1.207 (-3.332 to 5.746)	9.078	0.600

Abbreviations: CI, confidence interval; CCA, complete-case analysis; MMRM, mixed models for repeated measures; MI, multiple imputation.

In the Nocturnal Trial, no analytic approach demonstrated a statistically significant effect. At 4 months, estimates ranged from -0.64 (95% CI: -4.13 to 2.85, p = 0.714) under CCA to -1.06 (95% CI: -5.28 to 3.16, p = 0.620) under MI. At 12 months, point estimates were positive (mean difference range: 1.10 to 1.26) but imprecise, with wide confidence intervals spanning zero and p-values > 0.10.

These results indicate that in the Daily Trial, methods that retained partially observed longitudinal data (MMRM, MI) produced treatment-effect estimates similar to or slightly larger than CCA, particularly at 12 months when missingness was greater. In contrast, the Nocturnal Trial showed small, uncertain effects regardless of method. Given these distributional challenges and the potential limitations of parametric mean-based inference, we next examined the WinP as a complementary, rank-based estimand.

#### 3.3.2. Win Probability

Win probability analyses paralleled the mean difference findings but produced attenuated effect sizes (Table 5).

**Table 5.** Win Probabilities in PHC Scores Over Time in the FHN Trials

Timepoint	Method (n)	Effect Estimate (95% CI)	95% CI Width	p-value
**Daily Trial**				
4 months	CCA (212)	0.601 (0.547 to 0.653)	0.106	<0.001
​	MMRM (222)	0.601 (0.548 to 0.652)	0.104	<0.001
12 months	CCA (191)	0.589 (0.521 to 0.654)	0.133	0.011
​	MMRM (222)	0.589 (0.524 to 0.650)	0.125	0.007
**Nocturnal Trial**				
4 months	CCA (81)	0.471 (0.354 to 0.591)	0.237	0.634
​	MMRM (84)	0.463 (0.354 to 0.576)	0.222	0.521
12 months	CCA (68)	0.505 (0.382 to 0.628)	0.246	0.937
​	MMRM (84)	0.510 (0.391 to 0.627)	0.236	0.864

Abbreviations: CI, confidence interval; CCA, complete-case analysis; MMRM, mixed models for repeated measures.

In the Daily Trial, WinP at 4 months was 0.60 by CCA (95% CI: 0.55 to 0.65, p < 0.001) and 0.60 by MMRM (95% CI: 0.55 to 0.65, p < 0.001), indicating a 60% probability that a participant receiving frequent dialysis would report a better PHC score than one on conventional dialysis. At 12 months, WinP estimates again favoured the frequent dialysis arm (CCA: 0.59, 95% CI: 0.52 to 0.65, p = 0.011; MMRM: 0.59, 95% CI: 0.52 to 0.65, p = 0.007), with both methods reaching statistical significance.

In the Nocturnal Trial, WinP estimates showed no consistent evidence of benefit. At 4 months, WinP estimates were below the null (0.47), with wide confidence intervals crossing 0.50. At 12 months, WinP estimates shifted above the null (0.51) but again remained imprecise and non-significant.

Taken together, these findings reinforce the modest but consistent benefit of frequent dialysis in the Daily Trial and the inconclusive results in the Nocturnal Trial. They also demonstrate the value of WinP as a distribution-free complement to mean differences. In the Daily Trial, WinP estimates confirmed the same direction of benefit but expressed it in a patient-centered probability scale: an approximately 59% to 60% chance of better PHC with frequent dialysis. In the Nocturnal Trial, where distributions were clearly non-normal and contained outliers, WinP estimates fluctuated around the null but remained imprecise, underscoring the instability of treatment effects when parametric assumptions are violated. In both trials, WinP offered an interpretable summary that was less sensitive to skewness, ceiling/floor effects, and extreme values than mean differences, thereby illustrating its potential as a robust complement in the analysis of PROM data.

### 3.4. Sensitivity Analyses

By 12 months, there were 15 deaths in the Daily Trial and 3 deaths in the Nocturnal Trial. Consistent with the prespecified sensitivity framework described in Methods (Section 2.3.1), analyses used a hierarchical composite win fraction outcome (death plus PHC change) with death ranked as the worst outcome (Table 6). Findings were directionally consistent with the primary WinP analysis. In the Daily Trial, composite WinP by CCA was 0.66 (95% CI: 0.58 to 0.72; p < 0.001) at 4 months and 0.61 (95% CI: 0.54 to 0.68; p = 0.004) at 12 months, while MMRM estimates were 0.66 (95% CI: 0.59 to 0.73; p < 0.001) and 0.62 (95% CI: 0.54 to 0.69; p = 0.002), respectively. In the Nocturnal Trial, composite WinP remained near the null under both methods (CCA: 0.49 at 4 months and 0.51 at 12 months; MMRM: 0.49 at 4 months and 0.52 at 12 months), with wide confidence intervals crossing 0.50 and no statistically significant differences. Overall, accounting explicitly for death-related, potentially informative missingness did not materially change inference.

**Table 6.** Sensitivity Analysis of Win Probability Using a Hierarchical Composite Outcome

Timepoint	Method (n)	Effect Estimate (95% CI)	95% CI Width	p-value
**Daily Trial**				
4 months	CCA (215)	0.657 (0.583 to 0.724)	0.142	<0.001
​	MMRM (228)	0.660 (0.587 to 0.726)	0.139	<0.001
12 months	CCA (206)	0.612 (0.536 to 0.684)	0.138	0.004
​	MMRM (228)	0.617 (0.542 to 0.687)	0.145	0.002
**Nocturnal Trial**				
4 months	CCA (82)	0.492 (0.357 to 0.627)	0.270	0.906
​	MMRM (85)	0.487 (0.361 to 0.615)	0.254	0.847
12 months	CCA (71)	0.509 (0.369 to 0.649)	0.280	0.900
​	MMRM (85)	0.519 (0.382 to 0.653)	0.271	0.794

Abbreviations: CI, confidence interval; CCA, complete-case analysis; MMRM, mixed models for repeated measures.

## 4. Discussion

In this re-analysis of the FHN Daily and Nocturnal Trials, we found that both the analytic strategy for handling missing data and the choice of estimand influenced the interpretation of PROM results. In the Daily Trial, frequent dialysis yielded modestly higher PHC scores across analytic approaches, with estimates from MMRM and MI similar or slightly larger than those from CCA. In the Nocturnal Trial, treatment effects were small and imprecise regardless of method, reflecting the combination of smaller sample size, greater heterogeneity, and higher attrition.

These estimates should also be interpreted in relation to the original FHN Daily Trial results. Frequent dialysis was associated with a favourable coprimary composite outcome of death or decrease in RAND-36 PHC score (hazard ratio, 0.70; 95% CI, 0.53 to 0.92), and the adjusted mean RAND-36 PHC score increased by 3.4 ± 0.8 points in the frequent dialysis group versus 0.2 ± 0.8 points in the conventional dialysis group (p = 0.004).^23^ In the present reanalysis, the 12-month point estimates from CCA, MMRM, and MI were similar in magnitude; however, MMRM yielded a slightly larger estimate and stronger statistical evidence than CCA at 12 months, while MI produced a similar estimate. This statistical context informs, but does not determine, whether the approximately 3-point PHC advantage is clinically meaningful.

The estimated PHC advantage for frequent dialysis in the Daily Trial was approximately 3 points. In the original trial, detectable increases of 4.6 to 5.0 points in the RAND-36 PHC score were described as clinically meaningful.^23^ Our 12-month estimates were below this benchmark, although the confidence intervals included values exceeding it. PHC scores have established prognostic relevance for hospitalization and mortality in dialysis populations; therefore, even modest differences may be meaningful in context.^9-11^ The value of MMRM and MI in this setting is therefore best understood as improving interpretability and precision in the presence of incomplete PROM data, rather than simply changing statistical significance.

These findings highlight two critical methodological considerations. First, reliance on CCA can introduce bias and statistical inefficiency in the presence of monotone dropout, a pattern we observed in both trials. Participants with poorer baseline health were more likely to discontinue follow-up, making missing completely at random implausible and raising the possibility that CCA estimates were upwardly biased, although our data cannot confirm the direction or magnitude of any such bias. Consistent with this concern, and with ∼22% missing PHC observations by 12 months, CCA also reduced power, widened confidence intervals, and diminished the ability to detect treatment effects relative to methods retaining partially observed data. MMRM and MI, which leverage partially observed outcomes under the MAR assumption, yielded modestly larger and more statistically efficient (i.e., with smaller p-values) treatment-effect estimates in this analysis. The moderate-to-strong correlations across timepoints support the use of longitudinal data that retain incomplete cases and incorporate repeated observed PHC values. However, these correlations should not be interpreted as proof that MAR held; rather, MAR remains an unverifiable working assumption under which MMRM and MI are preferable to CCA.

Beyond the handling of missing data, a second consideration is the choice of estimand itself, which shapes how treatment effects are quantified and interpreted. PROMs such as the RAND-36 are prone to floor and ceiling effects, limited responsiveness in healthier subgroups, and skewed distributions.^31-33^ These features undermine the validity of parametric methods and conventional standardized effect sizes.^19^ Although widely used, standardized effect sizes (e.g., Cohen’s d) are rarely accompanied by confidence intervals and rely on distributional assumptions that are seldom satisfied in practice.^34,35^ Anderson et al (2022) have underscored these shortcomings, noting that confidence intervals are often omitted even under assumed normality and become especially problematic in the presence of missing data.^35^ By contrast, WinP allows construction of confidence intervals without relying on normality, offering a more robust alternative for quantifying treatment effects in PROMs with skewness, bounded scales, or missingness.^20,21^

Several limitations should be acknowledged. FHN eligibility likely selected a relatively healthier and more treatment-tolerant segment of the dialysis population, particularly individuals able and willing to attend frequent in-centre dialysis; therefore, external validity to frailer or less mobile patients may be limited. RAND-36 follow-up was also available at only two post-baseline visits (4 and 12 months), limiting temporal resolution of recovery trajectories and reducing ability to characterize nonlinear changes over time.

Our analyses further assumed MAR, which cannot be verified empirically. Although observed PHC scores were correlated over time and therefore provided information for longitudinal models, missing not at random mechanisms remain possible, especially given the high attrition rates and the likelihood that dropout was related to deteriorating health status. We also did not incorporate auxiliary variables beyond treatment arm, timepoint, centre, and baseline PHC score. If other unmeasured variables predicted both PHC and dropout, residual bias could persist. Moreover, we did not conduct formal missing not at random sensitivity analyses (e.g., pattern-mixture models or selection models) to assess departures from MAR. Finally, WinP was analyzed here as a secondary, post hoc estimand. The original FHN protocols specified a rank-based nonparametric analysis of the coprimary composite outcome at 12 months, using a log-rank test without covariate adjustment^23,24^; while valid for hypothesis testing, that framework does not directly provide adjusted effect-size estimates with confidence intervals and is not designed for repeated measures modeling across visits.^36^ WinP as labeled and parameterized in this re-analysis was not the original primary estimand. Accordingly, these results should be interpreted as exploratory.

Nonetheless, the modest improvements observed in the Daily Trial remain clinically relevant, given the established predictive value of PHC scores for hospitalization and mortality, while the lack of consistent effects in the Nocturnal Trial underscores the challenges of smaller, more heterogeneous cohorts. Taken together, these findings reinforce the need for pre-specified analytic strategies, including MMRM or MI as default approaches and complementary use of WinP alongside mean differences, to improve the transparency and interpretability of PROM results in dialysis trials.

## 5. Conclusions

Handling of missing data and choice of estimand can substantially alter the interpretation of PROM results in dialysis trials. Methods that retain incomplete cases, such as MMRM or MI, offer a theoretically preferable alternative to CCA when MAR is plausible. For skewed or bounded PROMs, WinP offers a nonparametric, patient-centered complement to mean differences. Future trials should pre-specify estimands, incorporate auxiliary variables, and adopt robust missing data strategies to strengthen validity and transparency. Given the high rates of PROM nonresponse in dialysis trials, analytic strategies that retain partially observed cases, leverage repeated measures, and incorporate sensitivity analyses should be prioritized. In this setting, MMRM and MI appeared more statistically efficient and less vulnerable to bias than CCA, reinforcing the need to move away from default reliance on CCA and toward routine use of likelihood- and imputation-based methods, while maintaining transparency and robustness checks to account for untestable assumptions.

## Supplemental Material

Supplemental Material - Methods for Addressing Missing Patient-Reported Outcome Data in Longitudinal Clinical Trials: A Secondary Analysis of the Frequent Hemodialysis Network Trials Supplemental material for Methods for Addressing Missing Patient-Reported Outcome Data in Longitudinal Clinical Trials: A Secondary Analysis of the Frequent Hemodialysis Network Trials by Victoria Zdunek, Guangyong Zou, Amit X. Garg and Pavel S. Roshanov in Canadian Journal of Kidney Health and Disease.

## Data Availability

The datasets analyzed during the current study are available in the NIDDK Central Repository, (Daily: https://repository.niddk.nih.gov/study/44, Nocturnal: https://repository.niddk.nih.gov/study/168).

## Appendix

### List of Abbreviations

PROM: Patient reported outcome measure
RAND-36: RAND 36-Item Health Survey
PHC: Physical health composite
MHC: Mental health composite
CCA: Complete-case analysis
WinP: Win probability
FHN: Frequent Hemodialysis Network
MMRM: Mixed models for repeated measures
MI: Multiple imputation
MAR: Missing at random
SD: Standard deviation

## References

[1] Weisbord SD Carmody SS Bruns FJ , et al. Symptom burden, quality of life, advance care planning and the potential value of palliative care in severely ill haemodialysis patients. Nephrology Dialysis Transplantation. 2003;18(7):1345-1352. doi:10.1093/ndt/gfg105. 12808172

[2] Jhamb M Weisbord SD Steel JL Unruh M . Fatigue in Patients Receiving Maintenance Dialysis: A Review of Definitions, Measures, and Contributing Factors. American Journal of Kidney Diseases. 2008;52(2):353-365. doi:10.1053/j.ajkd.2008.05.005. PMC2582327 18572290

[3] Finkelstein FO Finkelstein SH . Depression in chronic dialysis patients: assessment and treatment. Nephrology Dialysis Transplantation. 2000;15(12):1911-1913. doi:10.1093/ndt/15.12.1911. 11096130

[4] Mujais SK Story K Brouillette J , et al. Health-related Quality of Life in CKD Patients: Correlates and Evolution over Time. Clinical Journal of the American Society of Nephrology. 2009;4(8):1293-1301. doi:10.2215/CJN.05541008. PMC2723973 19643926

[5] Mapes DL Bragg-Gresham JL Bommer J , et al. Health-related quality of life in the Dialysis Outcomes and Practice Patterns Study (DOPPS). American Journal of Kidney Diseases. 2004;44:54-60. doi:10.1053/j.ajkd.2004.08.012. 15486875

[6] Tong A Manns B Hemmelgarn B , et al. Establishing Core Outcome Domains in Hemodialysis: Report of the Standardized Outcomes in Nephrology–Hemodialysis (SONG-HD) Consensus Workshop. American Journal of Kidney Diseases. 2017;69(1):97-107. doi:10.1053/j.ajkd.2016.05.022. PMC5369351 27497527

[7] Stevens PE Ahmed SB Carrero JJ , et al. KDIGO 2024 Clinical Practice Guideline for the Evaluation and Management of Chronic Kidney Disease. Kidney International. 2024;105(4):S117-S314. doi:10.1016/j.kint.2023.10.018. 38490803

[8] Ware JE Sherbourne CD . The MOS 36-item short-form health survey (SF-36). I. Conceptual framework and item selection. Med Care. 1992;30(6):473-483. 1593914

[9] Lowrie EG Curtin RB LePain N Schatell D . Medical outcomes study short form-36: a consistent and powerful predictor of morbidity and mortality in dialysis patients. American Journal of Kidney Diseases. 2003;41(6):1286-1292. doi:10.1016/S0272-6386(03)00361-5. 12776282

[10] Kalantar-Zadeh K Kopple JD Block G Humphreys MH . Association Among SF36 Quality of Life Measures and Nutrition, Hospitalization, and Mortality in Hemodialysis. Journal of the American Society of Nephrology. 2001;12(12):2797-2806. doi:10.1681/ASN.V12122797. 11729250

[11] Painter P Carlson L Carey S Paul SM Myll J . Physical functioning and health-related quality-of-life changes with exercise training in hemodialysis patients. American Journal of Kidney Diseases. 2000;35(3):482-492. doi:10.1016/S0272-6386(00)70202-2. 10692275

[12] Bell ML Floden L Rabe BA , et al. Analytical approaches and estimands to take account of missing patient-reported data in longitudinal studies. PROM. 2019;10:129-140. doi:10.2147/PROM.S178963. PMC6489631 31114411

[13] Bell ML Kenward MG Fairclough DL Horton NJ . Differential dropout and bias in randomised controlled trials: when it matters and when it may not. BMJ. 2013;346(jan21 1):e8668. doi:10.1136/bmj.e8668. PMC4688419 23338004

[14] Little RJA Rubin DB . Statistical Analysis with Missing Data. 1st ed. Wiley; 2002. 10.1002/9781119013563.

[15] Austin PC White IR Lee DS Van Buuren S . Missing Data in Clinical Research: A Tutorial on Multiple Imputation. Canadian Journal of Cardiology. 2021;37(9):1322-1331. doi:10.1016/j.cjca.2020.11.010. PMC8499698 33276049

[16] White IR Horton NJ Carpenter J Statistics RIMAS Pocock SJ . Strategy for intention to treat analysis in randomised trials with missing outcome data. BMJ. 2011;342(feb07 1):d40. doi:10.1136/bmj.d40. PMC3230114 21300711

[17] Fisher WP Cano SJ , eds. Person-Centered Outcome Metrology: Principles and Applications for High Stakes Decision Making. Springer International Publishing; 2023. 10.1007/978-3-031-07465-3.

[18] Stauder R Lambert J Desruol-Allardin S , et al. Patient-reported outcome measures in studies of myelodysplastic syndromes and acute myeloid leukemia: Literature review and landscape analysis. Eur J Haematol. 2020;104(5):476-487. doi:10.1111/ejh.13389. PMC7217037 31985078

[19] Page P . Beyond statistical significance: clinical interpretation of rehabilitation research literature. Int J Sports Phys Ther. 2014;9(5):726-736. PMC4197528 25328834

[20] Zou G Smith EJ Zou L Qiu SF Shu D . A rank-based approach to design and analysis of pretest-posttest randomized trials, with application to COVID-19 ordinal scale data. Contemporary Clinical Trials. 2023;126:107085. doi:10.1016/j.cct.2023.107085. 36657521

[21] Zou G Zou L Choi YH . Distribution-Free Approach to the Design and Analysis of Randomized Stroke Trials With the Modified Rankin Scale. Stroke. 2022;53(10):3025-3031. doi:10.1161/strokeaha.121.037744. 35975666

[22] Zou G Qui SF Zou J Smith ED Choi YH Bi Y . Rank-based methods for estimating landmark win probability in longitudinal randomized controlled trials with missing data. arXiv. 2026. doi:10.48550/ARXIV.2603.12454. Preprint posted online.

[23] In-Center Hemodialysis Six Times per Week versus Three Times per Week. N Engl J Med. 2010;363(24):2287-2300. doi:10.1056/NEJMoa1001593 PMC3042140 21091062

[24] Rocco MV Daugirdas JT Greene T , et al. Long-term Effects of Frequent Nocturnal Hemodialysis on Mortality: The Frequent Hemodialysis Network (FHN) Nocturnal Trial. American Journal of Kidney Diseases. 2015;66(3):459-468. doi:10.1053/j.ajkd.2015.02.331. PMC4549208 25863828

[25] Rocco MV Lockridge RSJ Beck GJ , et al. The effects of frequent nocturnal home hemodialysis: the Frequent Hemodialysis Network Nocturnal Trial. Kidney international. 2011;80(10):1080-1091. doi:10.1038/ki.2011.213. PMC3569086 21775973

[26] Fairclough DL . Design and Analysis of Quality of Life Studies in Clinical Trials. Chapman and Hall/CRC; 2010. doi:10.1201/9781420061185.

[27] Young R Johnson DR . Handling Missing Values in Longitudinal Panel Data With Multiple Imputation. J Marriage Fam. 2015;77(1):277-294. doi:10.1111/jomf.12144. PMC4477955 26113748

[28] Bell ML Horton NJ Dhillon HM Bray VJ Vardy J . Using generalized estimating equations and extensions in randomized trials with missing longitudinal patient reported outcome data. Psycho-Oncology. 2018;27(9):2125-2131. doi:10.1002/pon.4777. 29802657

[29] Zou G Zou L Qiu S . Parametric and nonparametric methods for confidence intervals and sample size planning for win probability in parallel-group randomized trials with Likert item and Likert scale data. Pharmaceutical Statistics. 2023;22(3):418-439. doi:10.1002/pst.2280 36524672

[30] Zou G Zou L . A Nonparametric Global Win Probability Approach to the Analysis and Sizing of Randomized Controlled Trials With Multiple Endpoints of Different Scales and Missing Data: Beyond O’Brien–Wei–Lachin. Statistics in Medicine. 2024;43(28):5366-5379. doi:10.1002/sim.10247. PMC11586912 39415652

[31] Delucchi KL Bostrom A . Methods for Analysis of Skewed Data Distributions in Psychiatric Clinical Studies: Working With Many Zero Values. AJP. 2004;161(7):1159-1168. doi:10.1176/appi.ajp.161.7.1159. 15229044

[32] Arslan J Benke K . Statistical Analysis of Ceiling and Floor Effects in Medical Trials. Applied Biosciences. 2023;2(4):668-681. doi:10.3390/applbiosci2040042.

[33] Marateb HR Mansourian M Adibi P Farina D . Manipulating measurement scales in medical statistical analysis and data mining: A review of methodologies. J Res Med Sci. 2014;19(1):47-56. PMC3963323 24672565

[34] Sullivan GM Feinn R . Using Effect Size-or Why the P Value Is Not Enough. J Grad Med Educ. 2012;4(3):279-282. doi:10.4300/JGME-D-12-00156.1. PMC3444174 23997866

[35] Anderson SF . Appropriately estimating the standardized average treatment effect with missing data: A simulation and primer. Behav Res. 2022;56(1):199-232. doi:10.3758/s13428-022-02043-8. 36547758

[36] Altman DG Bland JM . Parametric v non-parametric methods for data analysis. BMJ. 2009;338(apr02 1):a3167. doi:10.1136/bmj.a3167. 19342403

