# Supplementary material for: Methods for Addressing Missing Patient-Reported Outcome Data in Longitudinal Clinical Trials: A Secondary Analysis of the Frequent Hemodialysis Network Trials
Source: Can J Kidney Health Dis. 2026 Sep 28;13:20543581261491677. doi: 10.1177/20543581261491677 (PMC13769911; doi:10.1177/20543581261491677)
Supplement: Supplementary file 1 [file sj-pdf-1-cjk-10.1177_20543581261491677.pdf]

Supplementary Material for Methods for Addressing Missing Patient-Reported Outcome Data in Longitudinal Clinical Trials: A Secondary Analysis of the Frequent Hemodialysis Network Trials

Figure S1a. Ethics Approval.

Western Research

Date: 21 January 2025

To: Dr Pavel Roshanov

Project ID: 126285

Review Reference: 2025-126285-103779

Study Title: Advancing Statistical Methods for Evaluating Symptom Burden and Clinical Outcomes in Hemodialysis Patients

Application Type: HSREB Initial Application

Review Type: Delegated

Date Approval Issued: 21/Jan/2025 15:16

REB Approval Expiry Date: 21/Jan/2026

Dear Dr Pavel Roshanov

The Western University Health Science Research Ethics Board (HSREB) has reviewed and approved the above mentioned study as described in the WREM application form, as of the HSREB Initial Approval Date noted above. This research study is to be conducted by the investigator noted above. **All other required institutional approvals and mandated training must also be obtained prior to the conduct of the study**.

Documents Approved:

Document Name	Document Type	Document Date	Document Version
FHN Analysis Protocol Jan 14 2025	Protocol	14/Jan/2025	1
CRF FHN Statistical Advances	Other Data Collection Instruments	15/Jan/2025	1
SF 36 Questionnaire	Other Data Collection Instruments	15/Jan/2025	1

Documents Acknowledged:

Document Name	Document Type	Document Date	Document Version

REB members involved in the research project do not participate in the review, discussion or decision.

The Western University HSREB operates in compliance with, and is constituted in accordance with, the requirements of the TriCouncil Policy Statement: Ethical Conduct for Research Involving Humans (TCPS 2); the International Conference on Harmonisation Good Clinical Practice Consolidated Guideline (ICH GCP); Part C, Division 5 of the Food and Drug Regulations; Part 4 of the Natural Health Products Regulations; Part 3 of the Medical Devices Regulations and the provisions of the Ontario Personal Health Information Protection Act (PHIPA 2004) and its applicable regulations. The HSREB is registered with the U.S. Department of Health & Human Services under the IRB registration number IRB 00000940.

Please do not hesitate to contact us if you have any questions.

Electronically signed by:

[redacted] 21/Jan/2025 15:16

Reason: I am approving this document

Note: This correspondence includes an electronic signature (validation and approval via an online system that is compliant with all regulations, See Electronic System Compliance Review)

Figure S1b. Continuing Ethics Approval.

Date: 12 January 2026

To: Dr Pavel Roshanov

Project ID: 126285

Review Reference: 2025-126285-116583

Study Title: Advancing Statistical Methods for Evaluating Symptom Burden and Clinical Outcomes in Hemodialysis Patients

Application Type: Continuing Ethics Review (CER) Form

Review Type: Delegated

Date Approval Issued: 12/Jan/2026 13:08

REB Approval Expiry Date: 21/Jan/2027

Dear Dr Pavel Roshanov,

The Western University Research Ethics Board has reviewed the application. This study, including all currently approved documents, has been re-approved until the expiry date noted above.

REB members involved in the research project do not participate in the review, discussion or decision.

Western University REB operates in compliance with, and is constituted in accordance with, the requirements of the Tri-Council Policy Statement: Ethical Conduct for Research Involving Humans (TCPS 2); the International Conference on Harmonisation Good Clinical Practice Consolidated Guideline (ICH GCP); Part C, Division 5 of the Food and Drug Regulations; Part 4 of the Natural Health Products Regulations; Part 3 of the Medical Devices Regulations and the provisions of the Ontario Personal Health Information Protection Act (PHIPA 2004) and its applicable regulations. The REB is registered with the U.S. Department of Health & Human Services under the IRB registration number IRB 00000940.

Please do not hesitate to contact us if you have any questions.

Electronically signed by:

[redacted] 12/Jan/2026 13:08

Reason: I am approving this document

Note: This correspondence includes an electronic signature (validation and approval via an online system that is compliant with all regulations).

Page 1 of 1

Table S1. Summary of Missing-Data Methods

Method	Description	Key assumption(s)	Strengths	Limitations
CCA	Includes only participants with PHC scores observed at all scheduled timepoints.	Missing data are MCAR; excluded participants are exchangeable with included participants.	Simple to implement and interpret.	Discards partially observed participants, reduces precision, and may introduce bias when missingness is related to observed health status or outcomes.
MMRM	Uses observed PHC scores and models repeated measurements within participants.	MAR conditioning on observed outcomes and covariates included in the model.	Retains participants with partial follow-up; likelihood-based approach commonly recommended for longitudinal RCT outcomes collected at fixed timepoints.	MAR is unverifiable; results depend on model specification and included covariates.
MI	Creates multiple plausible values for missing PHC scores, analyzes each completed dataset, and combines estimates using Rubin's rules.	MAR given the variables in the imputation model.	Retains incomplete cases and reflects uncertainty about missing values; flexible when auxiliary variables are available.	Depends on correct specification of the imputation model and inclusion of relevant predictors of missingness and outcome.

Footnote: Abbreviations: CCA, complete case analysis; PHC, Physical Health Composite; MCAR, missing completely at random; MMRM, mixed models for repeated measures; MAR, missing at random; RCT, randomized controlled trial; MI, multiple imputation.

CJKHD-26-0027.R1

Résumé

Contexte : Il est fréquent dans les essais longitudinaux portant sur hémodialyse que des données soient manquantes dans les résultats rapportés par les patients, et que les preuves empiriques pour guider leur analyse et leur interprétation soient insuffisantes.

Objectif : Comparer différentes approches pour l'analyse et l'interprétation des résultats rapportés par les patients dans les essais cliniques du Frequent Hemodialysis Network (FHN).

Conception : Analyse secondaire de deux essais contrôlés randomisés multicentriques.

Cadre : Des centres d'hémodialyse et programmes d'hémodialyse à domicile en Amérique du Nord.

Sujets : Des participants aux essais *Daily* (n = 245) et *Noctural* (n = 87) du FHN.

Mesures : Scores composites de santé physique du RAND-36 à l'inclusion, puis à 4 et 12 mois.

Méthodologie : Les différences moyennes de scores à 12 mois ont été estimées à l'aide d'une analyse des cas complets (ACC), d'un modèle mixte pour mesures répétées (MMRM) et de l'imputation multiple (IM). Afin d'assouplir l'hypothèse de normalité et de faciliter l'interprétation des différences moyennes, des analyses fondées sur les rangs ont été réalisées pour estimer la probabilité de supériorité (*WinP*), soit la probabilité qu'un sujet traité obtienne à 12 mois un meilleur score qu'un sujet témoin.

Résultats : Des données complètes aux deux périodes post-randomisation étaient disponibles pour 74 % à 75 % des sujets. Dans l'essai *Daily*, les estimations de la différence moyenne à 12 mois (IC à 95 %) étaient de 3,04 (0,66-5,42; p = 0,012) avec le MMRM, de 2,97 (0,42-5,52; p = 0,023) avec l'IM et de 2,76 (0,31-5,21; p = 0,027) avec l'ACC. Les estimations de la *WinP* étaient de 0,59 (0,52-0,65; p = 0,007) avec le MMRM et de 0,59 (0,52-0,65; p = 0,011) avec l'ACC. Dans l'essai *Noctural*, les effets du traitement étaient faibles, imprécis et non statistiquement significatifs, quelle que soit la méthode utilisée.

Limites : Il s'agissait d'une analyse secondaire avec analyses exploratoires fondées sur les rangs.

Conclusion : Des approches intégrant les sujets dont les données sont partiellement disponibles pourraient s'avérer plus efficaces que les analyses de cas complets. L'analyse de la probabilité de

supériorité (*WinP*) a offert une solution de rechange aux différences moyennes dans les scores de mesure de résultats rapportés par les patients.
